# Zooplankton communities in the Drake Passage through environmental boundaries: a snapshot of 2010, early spring

**DOI:** 10.7717/peerj.7994

**Published:** 2019-11-07

**Authors:** Andrey A. Vedenin, Eteri I. Musaeva, Daria N. Zasko, Alexander L. Vereshchaka

**Affiliations:** Laboratory of Plankton Communities Structure and Dynamics, P.P. Shirshov Institute of Oceanology, Moscow, Russia

**Keywords:** Spatial distribution of zooplankton, Zooplankton communities, Drake passage

## Abstract

**Background:**

Spatial distribution of zooplankton communities influenced by various environmental factors is always important for understanding pelagic ecosystems. The area of the Drake Passage (Southern Ocean) is of particular interest owing to the high spatial and temporal variability of hydrological parameters affecting marine fauna. This study provides a survey of zooplankton composition and spatial distribution along a transect in the Drake Passage sampled during the 31th Cruise of RV “*Akademik Sergey Vavilov*” in November, 2010. The main aim was to trace the main regularities in spatial zooplankton structure and its relationships with the environmental parameters.

**Methodology:**

A total of 43 vertical hauls from the surface to 1,000 m depth were made at 13 stations using the Juday plankton net. 60 taxa were recorded, abundance and biomass of each were assessed. Environmental parameters including temperature, salinity, depth, horizontal distance between stations and surface chlorophyll concentration were tested as environmental factors possibly explaining plankton distribution.

**Results:**

Higher zooplankton abundance and biomass with lower diversity were observed near the Polar Front. Cluster analysis revealed five different groups of zooplankton samples, four of which were arranged mostly by depth. Along the transect within the 1,000 m depth range, the qualitative taxonomical composition differed significantly with depth and to some extent differed also among horizontal hydrological regimes, while the quantitative structure of the communities (abundance of taxa) was mainly determined by depth. Plankton assemblages within the upper 300-m layer depended on hydrological fronts. Abundance of dominant taxa as well as total zooplankton abundance showed a clear correlation with depth, salinity and surface chlorophyll concentration. Some taxa also showed correlations with temperature and latitude. Between the stations the similarity in zooplankton structure was clearly dependent on the distance among them which indicates an importance of latitudinal gradient. Surface chlorophyll concentration was not correlated with zooplankton biomass, which can be explained by the uncompleted seasonal migrations of zooplankton from deeper waters in early spring.

## Introduction

The role of various environmental parameters on plankton distribution including light, depth, temperature, water masses, etc. is extensively studied throughout the World Ocean (Aoki, Komatsu & Hwang, 1999; Hays, Richardson & Robinson, 2005; Longhurst, 1976; Labat et al., 2009; Lebourges-Dhaussy et al., 2009; Lucas et al., 2014). Zooplankton composition and distribution varies significantly over vertical and horizontal gradients (Vinogradov, 1968; Vereshchaka et al., 2016; Vereshchaka et al., 2017). Some species are restricted for certain depths, while others are known to make extensive diurnal, ontogenetic, or seasonal vertical migrations (Vinogradov, 1968; Longhurst, 1976; Taki, Hayashi & Naganobu, 2005; Tanimura et al., 2008; Cisewski et al., 2010). Specifically, in the spring many species arise to the surface water layers for feeding and reproduction in temperate, subpolar, and polar areas (Gliwicz, 1986; Żmijevska, 1987; Lampert, 1989; Park & Ferrari, 2009). Therefore, season and time of day are potentially strong factors influencing the vertical distribution of zooplankton. Another presumably significant factor is biogeographical location, linked to changes in zooplankton communities across horizontal boundaries. This is particularly important in the Southern Ocean, where the complex of different longitudinally arranged water masses, currents and fronts forms the Antarctic Circumpolar Current (Sokolov & Rintoul, 2009; Constable et al., 2014).

Antarctic Circumpolar Current (ACC) is composed of several jets and related hydrological fronts, which are known to act as significant boundaries for plankton communities (Pakhomov, Perissinotto & McQuaid, 1994; Pollard et al., 2002; Smetacek et al., 2002). The basic recognized hydrological fronts include the Subtropical Front (STF), sometimes termed as the northern boundary of the Southern Ocean, the Subantarctic Front (SAF), the Polar Front (PF) and the Southern Front (SF) (reviewed and summarized by Orsi, Whitworth & Nowlin, 1995). Areas between the fronts are referred to as the Subantarctic zone (SAZ, north from SAF), the Polar Front zone (PFZ, between SAF and PF) and the Antarctic zone (AZ, south from PF) (e.g., Demidov, Mosharov & Gagarin, 2012). In the narrowest area of the Southern Ocean, the Drake Passage, the overall structure of the ACC is simplified with some jets merged, forming fewer “superjets” (Olbers et al., 2004; Sokolov & Rintoul, 2009; Tarakanov & Gritsenko, 2018). This is the most dynamic area of the Southern Ocean, rich in temporal meanders and eddies. The structure of the ACC in the Drake Passage varies in number of jets recorded in different seasons (Olbers et al., 2004; Tarakanov & Gritsenko, 2018). However, the existence of fundamental ACC structures such as the SAF and the PF remains substantially stable within the Drake Passage (Orsi, Whitworth & Nowlin, 1995; Olbers et al., 2004; Tarakanov & Gritsenko, 2018).

First zooplankton observations in the Drake Passage area were collected during the *Discovery Investigations* in 1920-s (Mackintosh, 1937). The study revealed high horizontal and vertical heterogeneity of zooplankton and its dependence on hydrological factors. However, no quantitative analyses were performed at that date. Some of the *Discovery* data were rescued and retreated recently with more comprehensive statistics (Mackey et al., 2012). A number of zooplankton studies based on net samples from the area of Drake Passage including those focused on spatial distribution in relation to hydrological parameters were recently published (Kulagin, 2010; Stupnikova & Vereshchaka, 2013; Stupnikova et al., 2018; Takahashi et al., 2010). However, integral and taxonomical characteristics of plankton communities including the vertical structure were not analyzed so far in relation to a number of environmental parameters within this highly dynamic area. Here we start a series of publications focused on spatial distribution of zooplankton communities and dominant zooplankton species in the Drake Passage to analyze their relation to various environmental factors including depth, temperature, salinity, surface chlorophyll and fronts position. The aim of this study was to assess the main regularities of spatial zooplankton distribution with a special focus on its relationships with environmental parameters in the early spring of 2010.

## Materials & Methods

**Figure 1:**
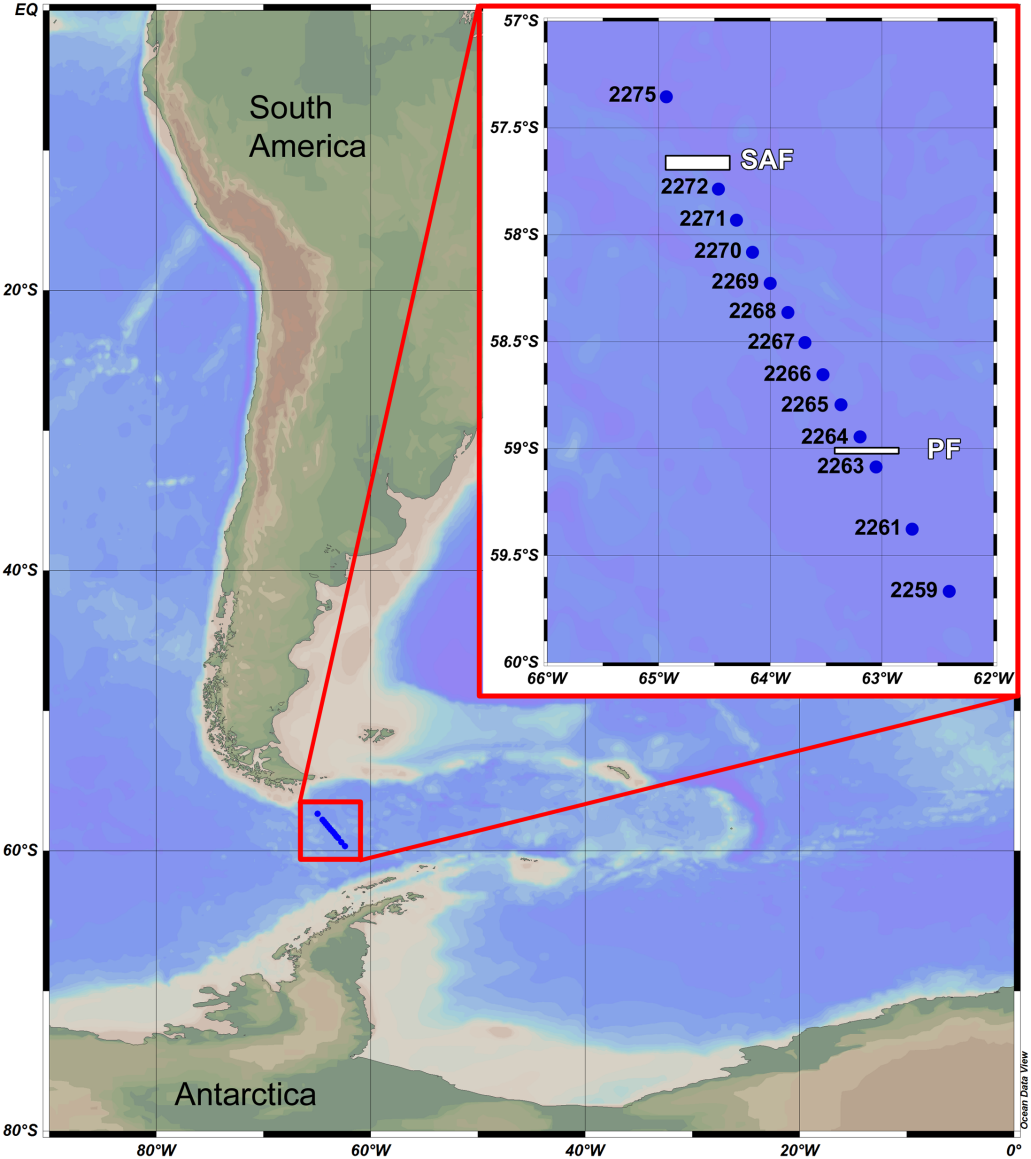
Study area and stations. SAF—position of Subantarctic current (=Subantarctic front); PF—position of South Polar current (=Polar front).

Samples were taken during the 31st Cruise of RV “*Akademik Sergey Vavilov*” in November 2010. A total of 13 stations were sampled using Juday plankton net with the mesh size of 0.18 mm and mouth area of 0.1 m^2^. Three to five vertical hauls sampled at different water layers (i.e., depth ranges) were taken at each station at a speed of 1 m/s. The net was equipped with the closing device. The depth range of hauls depended on hydrological gradients indicated by CTD-sensor at the same stations prior to biological sampling. Study area with stations and main fronts is shown in Fig. 1, other details, including the calculated filtered water volume are presented in Table 1.

All samples were fixed with 4% formalin and later sorted in laboratory by hand. All animals were identified to the lowest possible taxonomical level. Larval stages of crustaceans (including copepodite stages of copepods) were also identified. Abundance and biomass were calculated to cubic meter; biomass was calculated on the basis of the body shape and size using coefficients described by Chislenko (1968). List of each taxon density, biomass and larval stage is shown in Table S1.

Taxonomic diversity was estimated using the Shannon–Wiener index and the Hurlbert rarefaction index for 100 individuals (ES100). Square root transformed density was used as a measure of species abundance. In addition to quantitative parameters, the presence/absence qualitative data were used. We chose two approaches to the sample analysis—analyzing separate water layers (further referred to as “samples”, Analysis 1) and analyzing the whole 0–300 m depth range (upper samples combined for each station, further referred to as “stations”, Analysis 2). Clusters were built using UPGMA method based on quantitative and qualitative Bray-Curtis similarity indices. The presence of community structure within the samples was identified by the Similarity profile analysis (SIMPROF). The results of the cluster analysis were verified by the Analysis of similarities (ANOSIM). Taxa responsible for differences between the clusters were revealed by the Similarity percentage routine (SIMPER). Relations of integral community characteristics and dominant taxa and larval stages distribution to environmental factors were estimated using Pearson correlation coefficients and Canonical correspondence analysis (CCA) (McCune, Grace & Urban, 2002). The environmental parameters included temperature, salinity, depth, horizontal distance between stations and surface chlorophyll concentration (Table 2). Chlorophyll amount was estimated from satellite imaging data taken from Aqua MODIS (level 3, 4-km resolution, https://oceancolor.gsfc.nasa.gov/), averaged over 1 month to 1 latitudinal × 2 longitudinal degrees rectangles for each station. The averaging was done separately for September, October and November. For November data, the coordinates of rectangles were built around each station (with the station coordinates in the geometric rectangle center). For October and September the rectangle coordinates were calculated according to monthly eastward waters shift of around 10 longitudinal degrees. Latitudinal shift was assessed for each square by the mean fronts position and jets direction (Tarakanov & Gritsenko, 2018).

**Table 1:** Station data with coordinates and layers depth ranges.

**Station**	**Latitude(S)**	**Longitude(W)**	**Day/month/year**	**Local time**	**Layerdepth range(m)**	**Volume filtered (m^3^)**
				2:10	0–45	4.50
**2259**	59.67	62.39	07.11.2010	2:00	45–173	12.70
				1:50	170–300	13.00
				3:00	297–1,000	70.30
						
				10:00	0–50	5.00
**2261**	59.38	62.72	07.11.2010	9:50	50–180	13.00
				9:40	180–300	12.00
						
				17:10	0–50	5.00
**2263**	59.09	63.04	07.11.2010	17:00	50–190	14.00
				16:50	190–300	11.00
						
				22:10	0–130	13.00
**2264**	58.95	63.20	07.11.2010	22:00	130–235	10.50
				21:50	235–300	6.50
						
				2:30	0–60	6.00
				2:20	60–180	12.00
**2265**	58.80	63.36	08.11.2010	2:10	180–250	7.00
				2:00	250–300	5.00
				3:40	296–1,000	70.40
						
				6:00	0–56	5.60
**2266**	58.66	63.52	08.11.2010	5:50	55–200	14.50
				5:40	200–300	10.00
						
				9:20	0–70	7.00
**2267**	58.51	63.69	08.11.2010	9:10	70–220	15.00
				9:00	220–300	6.00
						
				13:10	0–50	5.00
**2268**	58.37	63.84	08.11.2010	13:00	50–200	15.00
				12:50	200–300	10.00
						
				17:15	0–50	5.10
**2269**	58.23	63.99	08.11.2010	17:00	50–200	15.00
				16:50	200–300	10.00
				21:20	0–50	5.00
**2270**	58.09	64.15	08.11.2010	21:10	50–200	15.00
				21:00	200–300	10.00
						
				2:20	0–55	5.50
**2271**	57.94	64.30	09.11.2010	2:10	55–160	10.50
				2:00	160–300	14.00
				3:20	296–1,000	70.40
						
				6:55	0–100	10.60
**2272**	57.79	64.46	09.11.2010	6:40	100–200	10.80
				6:30	200–300	10.90
						
				20:40	0–120	12.00
**2275**	57.36	64.93	09.11.2010	20:30	120–210	9.00
				20:20	210–300	9.00

**Table 2:** Surface chlorophyll *a* values calculated for different months, mean values of temperature and salinity at each station. Temperature and salinity are calculated for the upper 300 m. Chlorophyll values are calculated for the surface.

**Stations**	2259	2261	2263	2264	2265	2266	2267	2268	2269	2270	2271	2272	2275
October Chlorophyll, mg m^−3^	0.200	0.135	0.138	0.131	0.126	0.120	0.118	0.117	0.116	0.113	0.110	0.112	0.119
November Chlorophyll, mg m^−3^	0.362	0.301	0.237	0.186	0.196	0.211	0.213	0.200	0.188	0.181	0.159	0.135	0.207
Mean temperature, °C	0.527	0.298	0.578	1.617	1.803	2.284	2.606	1.937	2.746	2.551	2.634	2.927	4.825
Mean salinity, PSU	22.99	33.95	33.94	33.97	33.95	33.99	34.02	33.96	34.01	34.00	34.00	34.01	34.11

Statistics were performed using Primer v6, Past 3, Surfer 15 and Microsoft Excel 2010 software (Clarke & Warwick, 2001; Hammer, Harper & Ryan, 2003).

## Results

### Hydrological setting

**Figure 2:**
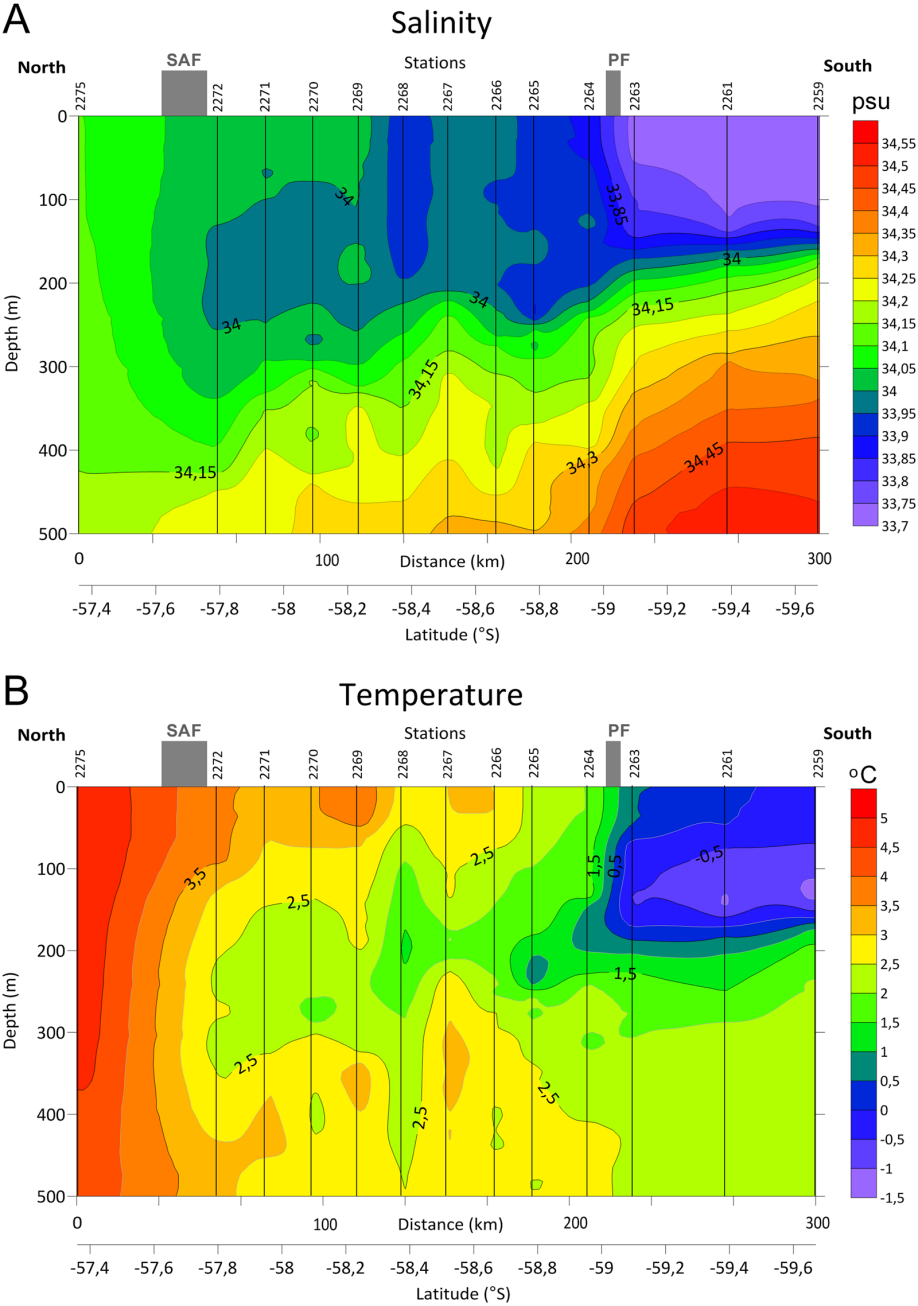
Distributions of Salinity (A) and Temperature (B) in upper 500 m layer along the transect with stations and main currents positions.

Distinct gradients of both temperature and salinity were detected along the transect between the stations 2275 and 2272 and north from the station 2263, where the SAF and the PF were located, respectively (Fig. 2, see also Tarakanov & Gritsenko, 2018). A steeper bathymetric gradient of both temperature and salinity was observed at three southern stations (2259, 2261 and 2263, Fig. 2). Station 2268 differed from the neighboring stations by lower salinity in the upper layer and by lower temperature in the ∼100–450 m depth range.

### Integral community characteristics

**Figure 3:**
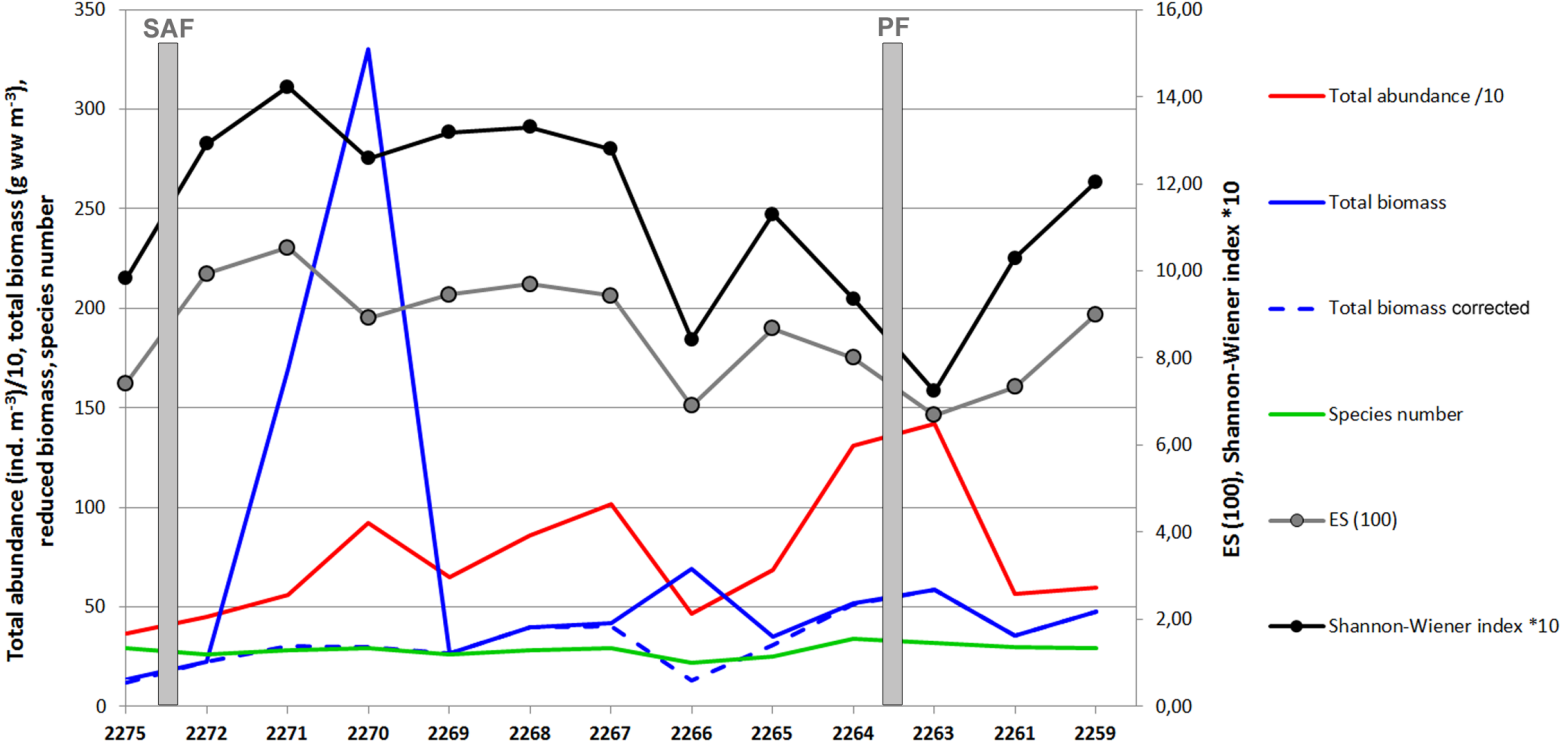
Values of total abundance, biomass, species number and diversity indices (Hurlbert rarefaction ES (100) and Shannon-Wiener index). Total abundance values are divided by 10; Shannon-Wiener index values are multiplied by 10.

A total of 60 zooplankton taxa (including 31 taxa of Copepoda) were recorded at 13 stations. Abundances and wet biomass values in samples varied from 6.6 ind m^−3^ and 0.75 mg m^−3^ (station 2271, the layer 296–1,000 m) to 4239 ind m^−3^ (station 2263, layer 0–50 m) and 1,883 mg m^−3^ (station 2270, layer 0–55 m). The values per station in the upper ∼300 m varied from 640 ind m^−3^ (station 2266) and 13.65 mg m^−3^ (station 2275) to 2,668 ind m^−3^ (station 2263) and 330 mg m^−3^ (station 2270). Prominent biomass peak was observed at station 2270 owing to large specimens of krill (*Thysanoessa* sp.) and fish larvae caught at in the layer 0–55 m (see Supplemental Information). After the krill and fish biomass was removed, the biomass trend became similar to the abundance trend with maximum values observed in the vicinity of the PF (Fig. 3).

The diversity values including both ES (100) and Shannon–Wiener indexes demonstrated clear decrease at station 2266 and near the PF zone (Fig. 3).

**Figure 4:**
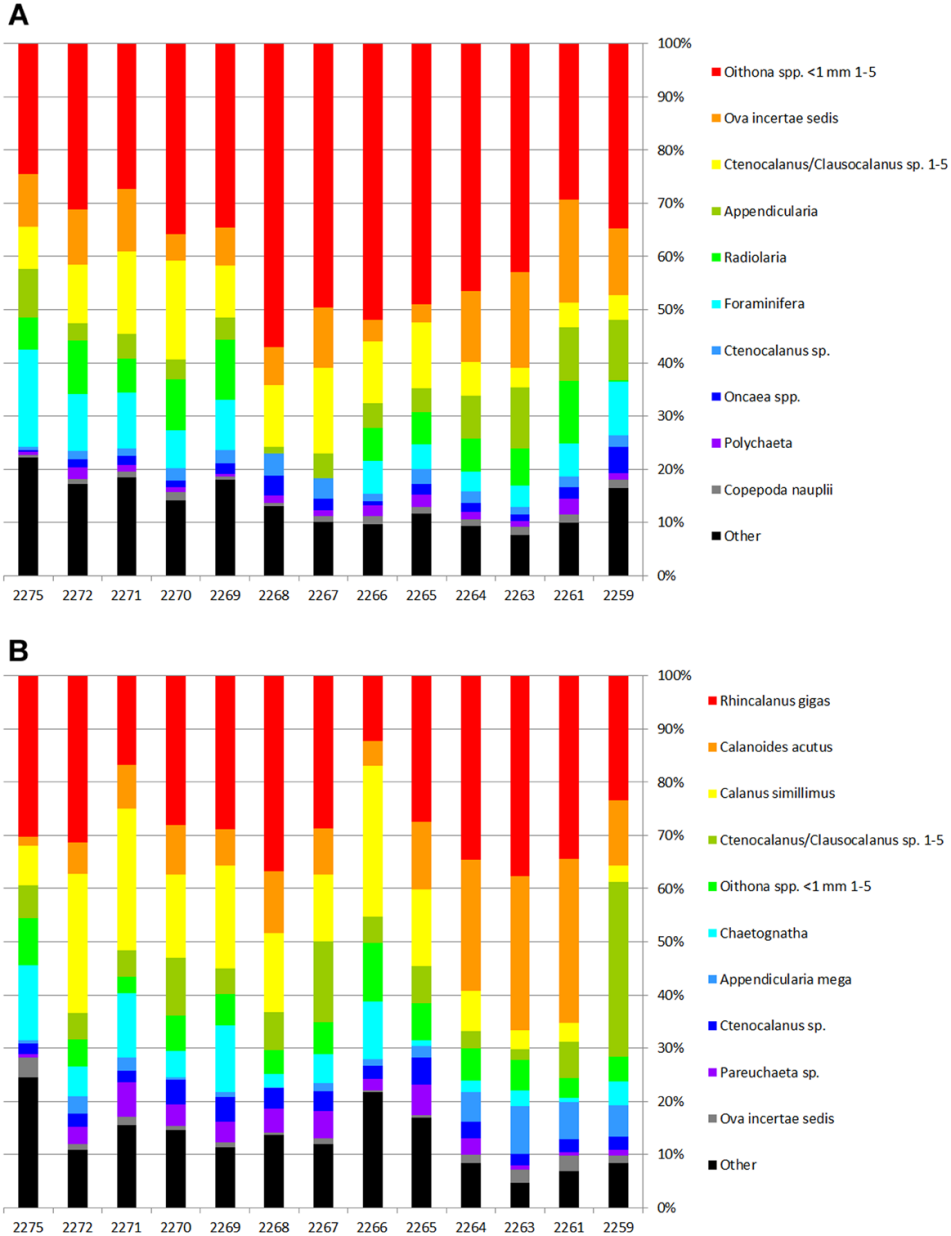
Proportions of the dominant taxa density (A) and biomass (B) at each station.

The most abundant taxa were *Oithona* sp. copepodites (up to 43% of total abundance) followed by ova of unidentified invertebrates, various stages of *Ctenocalanus* sp. and *Clausocalanus* sp., young unidentified copepodites of Copepoda, Appendicularia, Radiolaria, and Foraminifera. Ten most abundant taxa represented 93% of the total abundance and biomass (Table 3, Fig. 4). Proportions of the main dominant taxa at each station are shown in Fig. 4.

**Table 3:** Abundance and biomass of ten dominant taxa. Mean values and standard deviation (SD) are shown. Numbers after the taxa manes indicate copepodite stage.

**Abundance (ind m^−3^)**			**Biomass (g ww m^−3^)**		
**Taxon**	**Mean**	**SD**	**Taxon**	**Mean**	**SD**
*Oithona* sp. <1 mm 1–5	524.64	281.77	*Rhincalanus gigas*	9.54	5.76
Ova incertae sedis	151.05	131.09	*Calanoides acutus*	4.89	5.07
*Ctenocalanus/Clausocalanus* sp. 1–5	124.78	61.35	*Calanus simillimus*	3.88	2.18
Appendicularia	91.72	84.29	*Ctenocalanus/Clausocalanus* sp. 1–5	2.69	2.86
Radiolaria	84.38	65.51	*Oithona* spp. <1 mm 1–5	1.77	0.76
Foraminifera	84.21	52.27	Chaetognatha	1.60	1.00
*Ctenocalanus* sp.	28.43	14.02	Appendicularia large	1.19	1.50
*Oncaea* spp.	25.39	15.24	*Ctenocalanus* sp.	1.01	0.46
Polychaeta	18.24	10.35	*Pareuchaeta* sp.	1.00	0.70
Copepoda nauplii	16.18	10.72	Ova incertae sedis	0.47	0.38

### Analysis of samples (Analysis 1, water layers approach)

**Figure 5:**
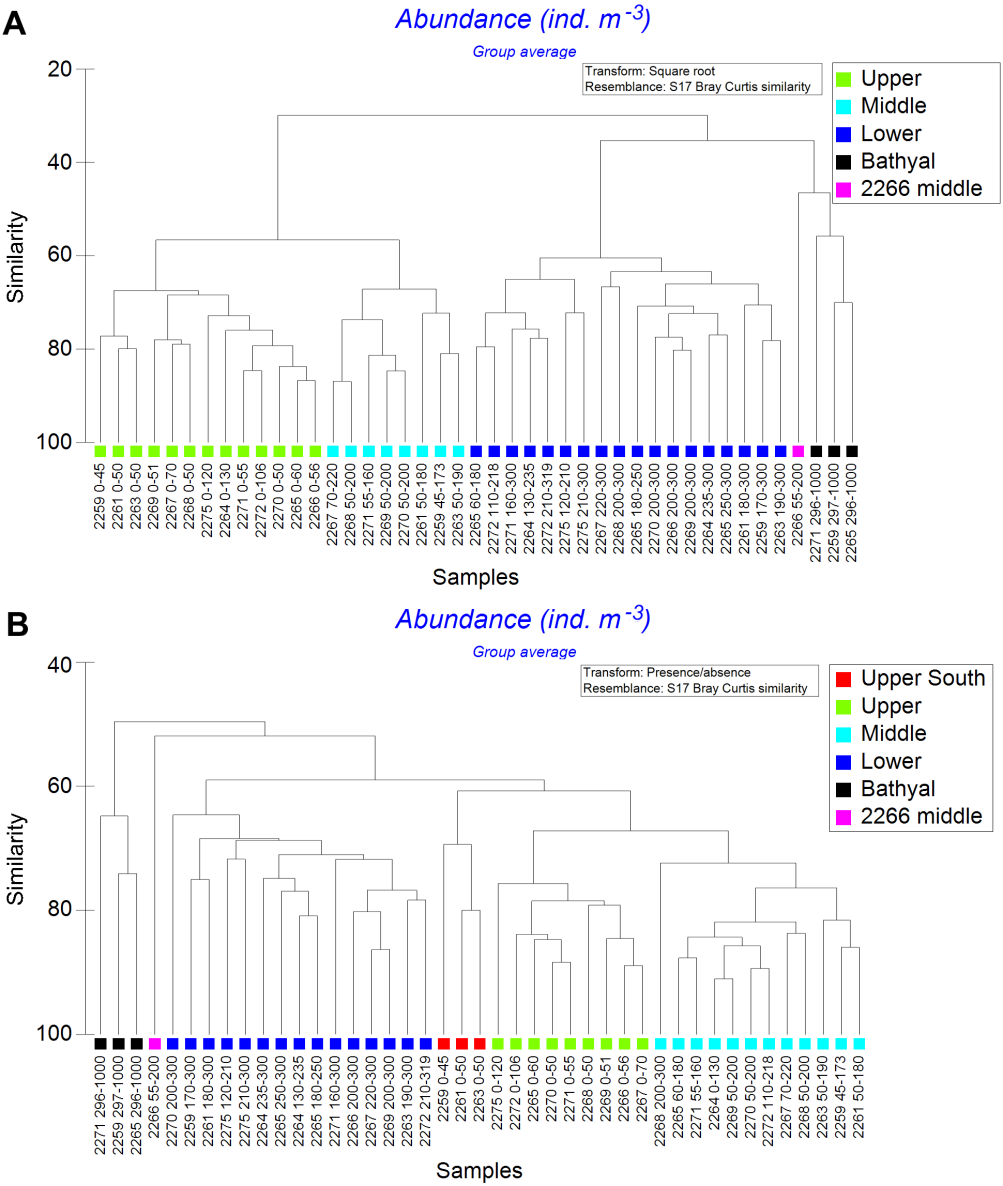
Cluster analysis of all samples using the Bray–Curtis similarity index. Color indicates station group distinguished by certain similarity level. A—square-root transformed data; B—qualitative presence/absence data. Sample coding: station number followed by depth range in meters.

**Figure 6:**
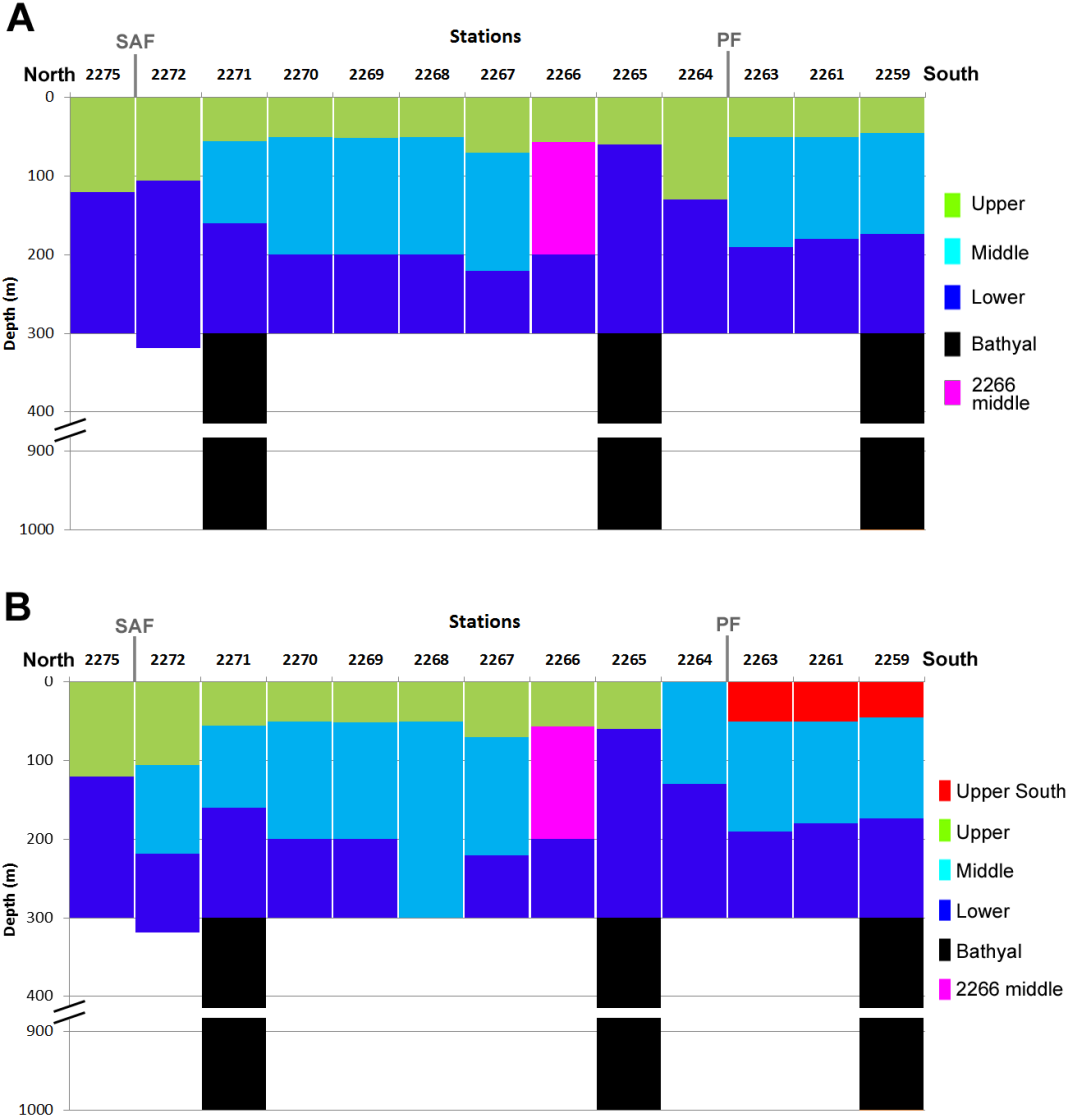
Vertical distribution of the revealed clusters along the transect. Colors as in Fig. 4. Approximate positions of SAF and PF are shown.

Cluster analysis of all samples revealed several distinct clusters corresponded to various water layers. Within the dendrogram based on quantitative square-root transformed data, four clusters named *Upper*, *Middle*, *Lower* and *Bathyal* were revealed (Fig. 5). A single sample (station 2266, the layer 55–200 m, named *2266 Middle*) was different from other stations (Fig. 5A) due to the absence of *Ctenocalanus* sp. and several other less common taxa (Supplemental Information). Clusters were divided by the similarity value of 57. In case of quantitative presence/absence data, the clusters were similar, with additional one named *Upper South* (Fig. 5B). Community composition was significantly different among all stations, SIMPROF-analysis demonstrated *π*-values >5.3 (with the mean *p*-value 0.01); sample statistic was not reliable in case of comparisons with *2266 Middle* cluster due to a single station in that cluster (Table 4). Vertical distribution of the revealed clusters along the transect is shown in Fig. 6.

Results obtained from square-root transformed data and from qualitative data were similar with the exception of three southern stations, located south from the PF, where the upper layers formed a separate cluster (Fig. 6). The *2266 Middle* layer samples were not associated with any visible hydrological gradients (Figs. 3 and 6). The northern-most stations (2275 and 2272), and stations located north off PF (2265 and 2264) consisted of only two clusters (*Upper* and *Lower*).

**Table 4:** Results of the ANOSIM analyses. Comparisons with *2266 Middle* station are removed from the table.

**Groups**	***R* Statistic**	**Significance (%)**	**Possible permutations**	**Actual permutations**	**% Shared taxa**
Square root overall transform, Bray–Curtis similarity					
*Upper-Middle*	0.79	0.001	203,490	999	76.0
*Upper-Lower*	0.999	0.001	206,253,075	999	64.6
*Upper-Bathyal*	1	0.002	560	560	48.7
*Middle-Lower*	0.919	0.001	1,562,275	999	56.3
*Middle-Bathyal*	1	0.006	165	165	52.5
*Lower-Bathyal*	0.994	0.001	1,330	999	58.8
Presence/absence overall transform, Bray–Curtis similarity					
*Upper South-Middle*	0.973	0.002	455	455	49.0
*Upper South-Lower*	0.977	0.001	816	816	42.2
*Upper South-Bathyal*	1	0.100	10	10	34.3
*Upper South-Upper*	0.975	0.005	220	220	56.3
*Middle-Lower*	0.643	0.001	17,383,860	999	68.4
*Middle-Bathyal*	0.993	0.002	455	455	52.4
*Middle-Upper*	0.85	0.001	293,930	999	66.0
*Lower-Bathyal*	0.905	0.001	816	816	61.1
*Lower-Upper*	0.985	0.001	1,307,504	999	61.0
*Bathyal-Upper*	1	0.005	220	220	45.0

Overall, the depth factor influenced species composition more significant than the latitude and the position in relation to hydrological fronts (Fig. 6). Particularly, mean Bray-Curtis similarity within each station was 39.68 ± 17.17 SD, whereas the similarity within each water layer was 66.99 ± 7.55 (in case of square-root transformed data). At the same time, certain latitudinal structure remained within some of the clusters, e.g., the subcluster of three stations south from PF (2259, 2261 and 2263) is clearly visible within *Upper*, *Middle* and *Lower* groups (Fig. 5A).

### Analysis of stations (Analysis 2, horizontal gradient approach)

**Figure 7:**
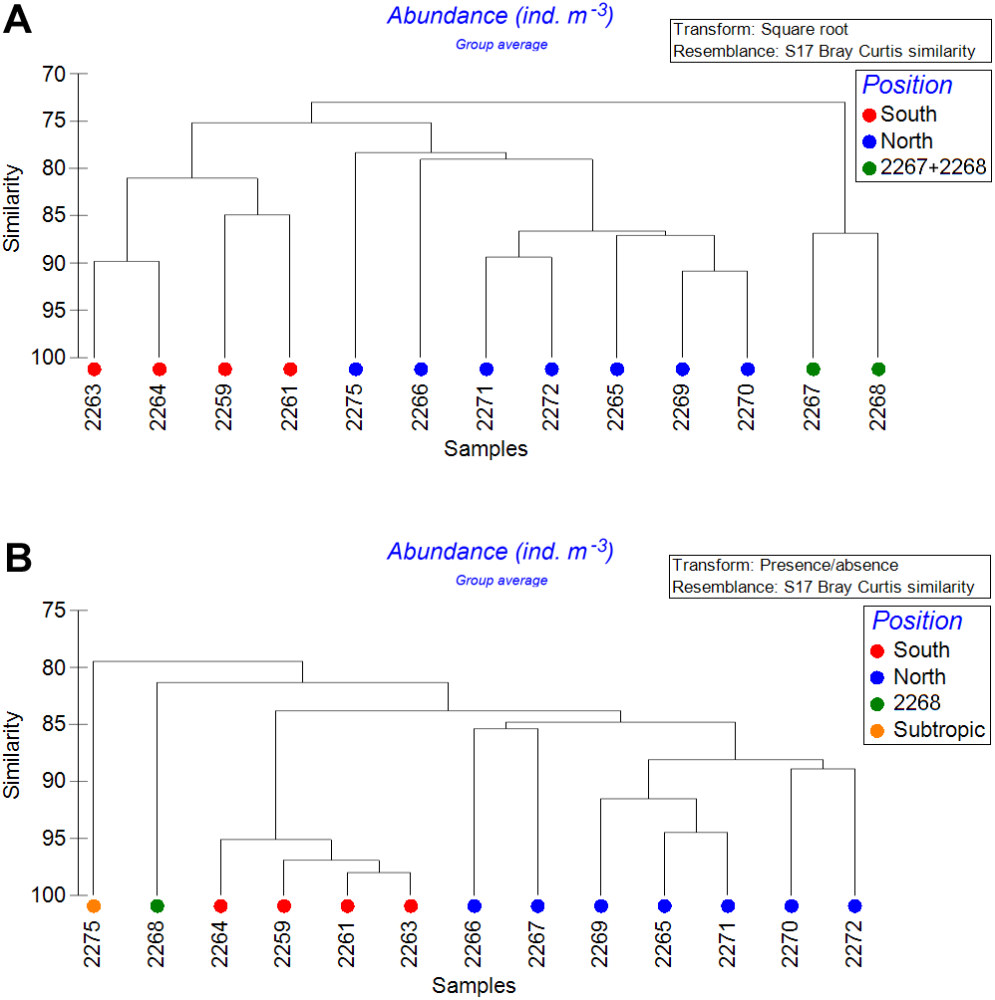
Cluster analysis of stations with upper 300 m layers combined using the Bray–Curtis similarity index. Color indicates station groups distinguished by certain similarity level. A—square-root transformed data, similarity level = 77; B—qualitative presence/absence data, similarity level = 83.

After combining the samples within the upper 300 m, the depth factor was removed from the analysis (Fig. 7). Both square-root transformed and quantitative data showed that four southernmost stations formed one cluster (*South*), while most of other stations formed another cluster (*North*). In addition, the square-root transformed data demonstrated a third cluster (stations 2267 and 2268). The quantitative data demonstrated two more clusters, each consisting of a single station: the northernmost station 2275, (*Subtropic*) and the station 2268 (Fig. 7). Clusters were divided based on Bray-Curtis similarity levels of 76% and 83% for the square-root-transformed and for the presence-absence data, respectively. Thus, the plankton assemblages within the upper 300-m layer showed dependence on hydrological fronts position, especially when the presence/absence data were analyzed.

### Comparison of zooplankton communities with environmental characteristics

**Figure 8:**
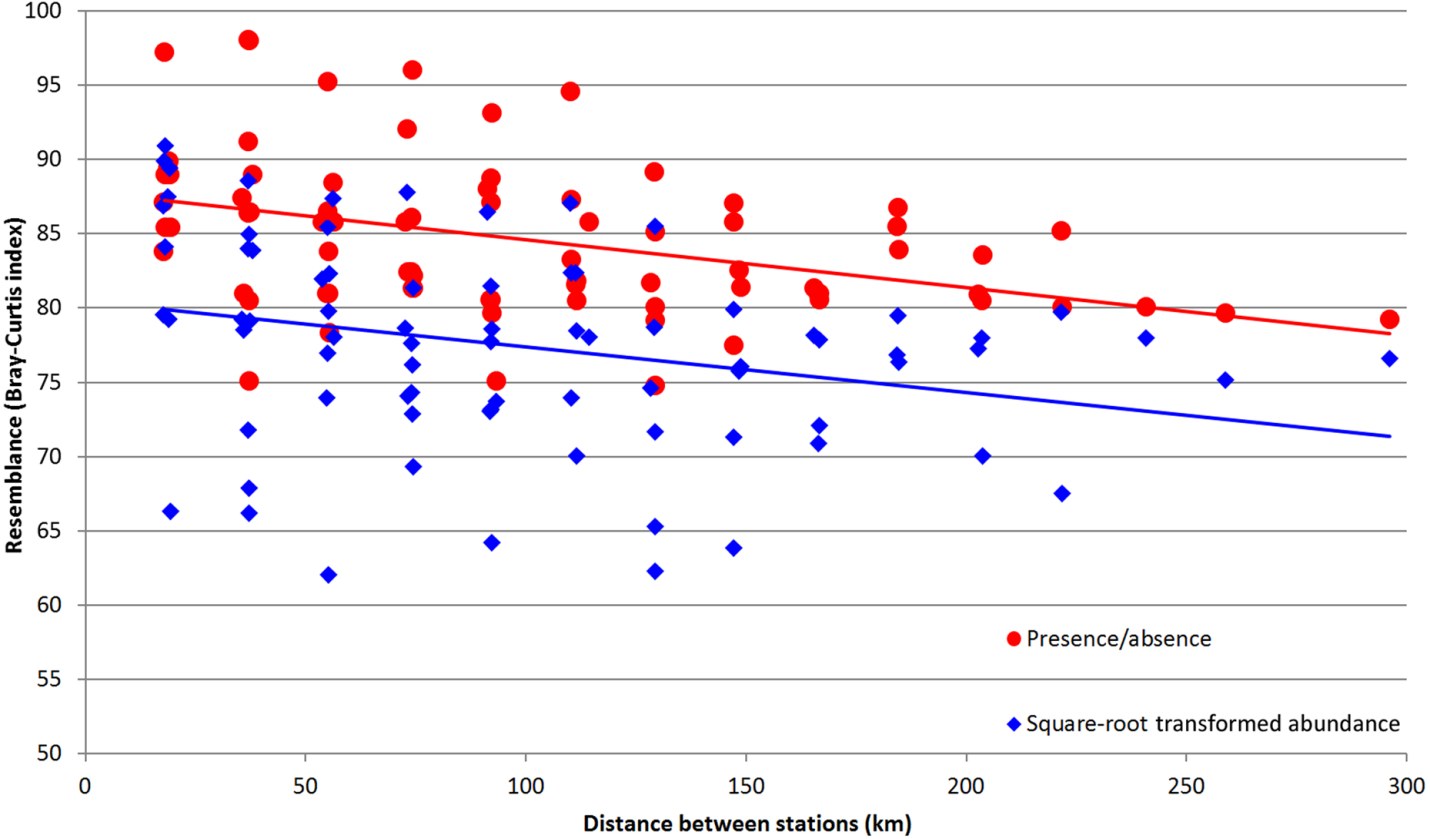
Values of Bray–Curtis similarity index by pairwise distance between the stations. Red dots indicate the similarity index calculated for presence/absence data; blue dots indicate the similarity index calculated for square-root transformed abundance data; lines of corresponding color indicate the linear trend.

Distinctive relation was found between the depth and total zooplankton abundances (Table 5). Abundances of certain taxa, including *Metridia curticauda*, *Lucicutia* sp. and *Gaetanus* sp. increased with depth, while abundances of pteropods, polychaetes, nauplii, *Calanus simillimus* and copepodites of *Oithona* sp. decreased with depth. Significant correlations, both positive and negative, were found between the chlorophyll concentrations and the abundances of several copepod species, larvae, Appendicularia and *Tomopteris* sp. (Table 5). The highest correlation values were observed when we used the chlorophyll data calculated for October for the entire upper 300-m layer. Integral community characteristics did not show any reliable correlation with chlorophyll except the total biomass, which was positive but not reliable (*p* = 0.087). A single taxon showed positive correlation with latitude (*Clausocalanus brevipes*) (Table 5). In addition, a clear linear dependence of Bray-Curtis similarity level on distance between the stations was observed (Fig. 8). The values of Pearson correlation were small (*R*-values −0.29 for square-root transformed abundance data and −0.40 for presence-absence data), but reliable (*p*-values 0.0094 and 0.0003, respectively). Despite the difference in mean similarity between the presence/absence and square-root-transformed abundance data, the linear trends of both sets demonstrated the same angles (Fig. 8).

**Table 5:** Pearson linear correlation between environmental parameters and abundances of community/taxa. Chlorophyll values were calculated for October, averaged over 1 × 2 degrees rectangles shifted westward by 10 degrees from the station longitude. Correlated pairs with *R* > 0.5 are shown.

**Environmental parameter**	**Community/taxon characteristic**	***R***	***p*(uncorr.)**
Depth	*Metridia curticauda*	0.78	9.67E−10
Depth	*Lucicutia* sp.	0.69	3.36E−07
Depth	Total abundance	−0.66	1.68E−06
Depth	*Oithona* sp. <1 mm stages 1–5	−0.61	1.45E−05
Depth	Calanidae gen.sp.2	0.55	0.0001
Depth	Ova	−0.55	0.0001
Depth	Polychaeta	−0.53	0.0002
Depth	Pteropoda	−0.53	0.0003
Depth	*Gaetanus* sp.	0.52	0.0004
Depth	Medusae	−0.51	0.0005
Depth	*Calanus simillimus*	−0.51	0.0005
Depth	Copepoda nauplii	−0.51	0.0005
Latitude	*Clausocalanus brevipes*	0.51	0.0004
Temperature	Larvae gen.sp.	−0.54	0.0002
Salinity	Appendicularia large	−0.62	7.93E−06
Salinity	Calanidae gen.sp.2	0.55	0.0001
Salinity	Ova	−0.53	0.0002
Salinity	Total abundance	−0.52	0.0003
Salinity	*Lucicutia* sp.	0.52	0.0003
Salinity	Appendicularia	−0.52	0.0003
Salinity	Medusae	−0.50	0.0006
Chlorophyll	*Metridia gerlachei*	0.95	5.79E−07
Chlorophyll	*Scaphocalanus* sp.	0.95	1.01E−06
Chlorophyll	*Calanus propinquus*	0.80	0.0010
Chlorophyll	*Oncaea* sp.	0.77	0.0021
Chlorophyll	Larvae gen.sp.	0.74	0.0041
Chlorophyll	Appendicularia large	0.69	0.0096
Chlorophyll	*Tomopteris* sp.	0.59	0.0338
Chlorophyll	*Aetideus armatus*	−0.57	0.0400
Chlorophyll	*Calanus simillimus*	−0.64	0.0200
Chlorophyll	Total biomass	0.49	0.0870

**Notes.**

Unreliable correlation between chlorophyll and total biomass is marked with grey.

We provided the SIMPER analysis between the station groups divided by the SAF and the PF (Table 6). Taxa responsible for most taxonomical dissimilarity between these boundaries were *Oithona* sp., *Ctenocalanus/Clausocalanus* copepodites, *Oncaea* sp. *Ctenocalanus* sp. and *Oithona frigida.* These taxa contributed >90% to the total dissimilarity between the stations.

**Table 6:** Combined results of SIMPER analysis of stations.

**Species**	**Average abundance (ind m^−3^)**			**Mean dissimilarity**	**Mean contribution**
	**SAZ**	**PFZ**	**AZ**		
*Oithona* sp. <1 mm stages 1–5	254.44	505.60	671.83	22.06	65.37
*Ctenocalanus/Clausocalanus* sp. stages 1–5	82.87	146.22	74.43	4.06	12.76
*Oncaea* sp.	3.27	22.53	41.36	2.21	6.40
*Ctenocalanus* sp.	5.33	29.63	32.53	1.70	4.95
*Oithona frigida*	2.90	17.18	5.45	0.78	2.45
*Calanus simillimus*	2.13	9.29	2.30	0.40	1.28
*Calanoides acutus*	0.60	3.79	8.47	0.40	1.18
*Clausocalanus laticeps*	0.30	7.84	0.07	0.39	1.25
*Rhincalanus gigas*	0.99	4.29	6.02	0.28	0.83
*Microcalanus pygmaeus*	2.23	4.11	5.62	0.24	0.74
*Scolecithricella minor*	1.47	5.09	2.66	0.20	0.64
*Metridia lucens*	5.23	4.66	4.68	0.15	0.46

**Notes.**

SAZ Subantarctic zone (station 2275) PFZ Polar Front zone (stations 2264–2272) AZ Antarctic zone (stations 2259–2263)

**Figure 9:**
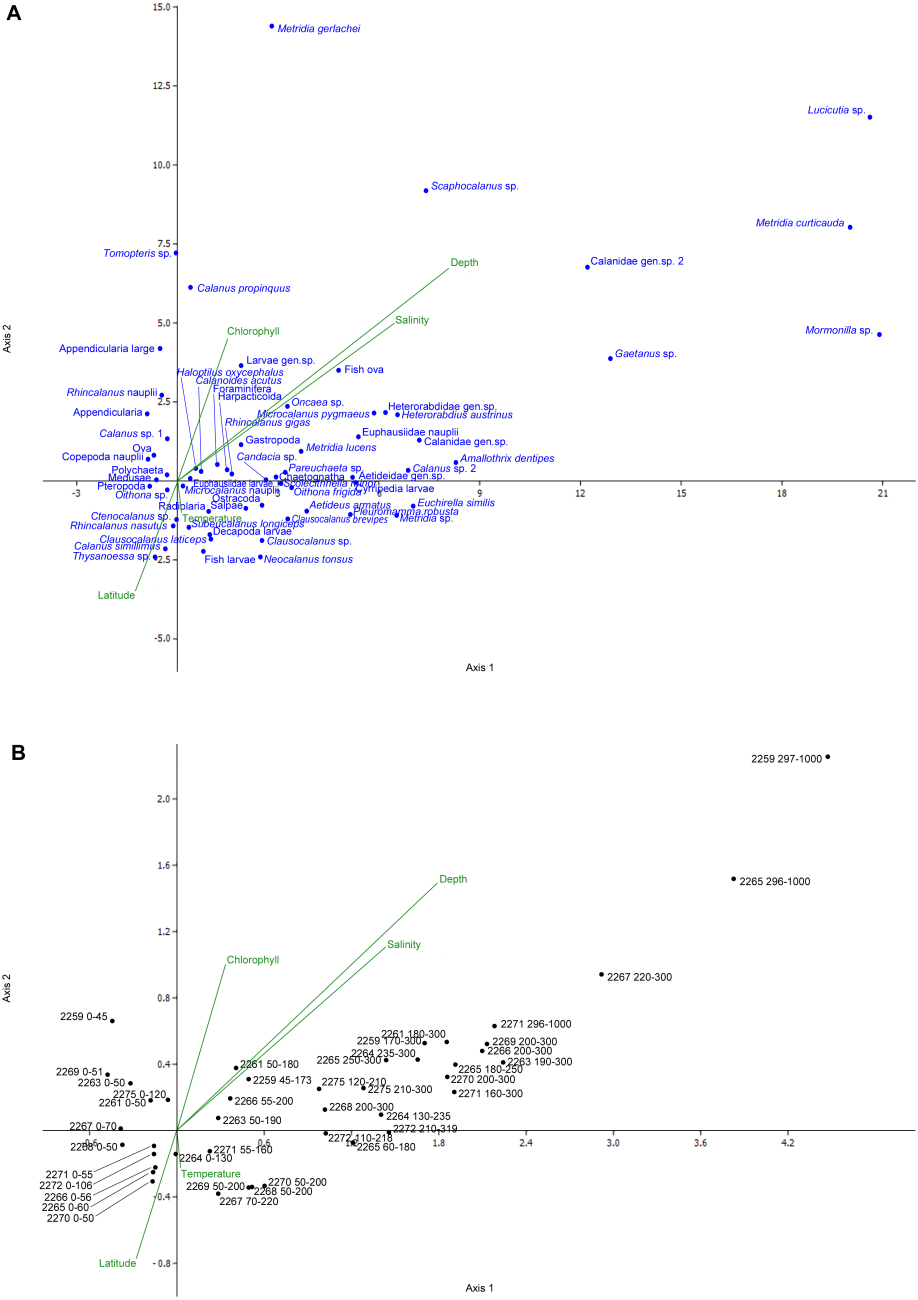
Canonical correspondence analysis (CCA) plot. A—species; B—samples; scales of the plots are equal. The overall significance level—0.246; CCA axis 1 significance level—0.174; CCA axis 2 significance level—0.046.

The most abundant taxa (including *Oithona* sp., *Ctenocalanus* sp., Appendicularia, Foraminifera, Radiolaria, for density values see Supplemental Information) were concentrated in the center of the CCA plot, demonstrating no significant deviations to either of environmental axes (Fig. 9A). Several less abundant species were located at plot margins, including three taxa found in the deepest layers (*Lucicutia* sp., *Mormonilla* sp., *Metridia curticauda*) and taxa found only at northern stations (e.g., *Rhincalanus nasutus*, Salpae). Several taxa depended on Chlorophyll axis (*Tomopteris* sp., *Calanus simillimus*) (Fig. 9A). Samples tended to arrange along the Depth axis rather than along the Latitude axis (Fig. 9B). Overall, the depth gradient was more significant than the latitude and other gradients (Figs. 9A and 9B).

## Discussion

### Zooplankton community abundance, biomass and diversity

Zooplankton abundance, biomass and biodiversity obtained in this study from the Drake Passage are in agreement with those reported in the previous investigations (Table 7) (Żmijevska, 1987; Ward et al., 2007; Mackey et al., 2012; Hosie et al., 2014). In particular, similar abundances were previously reported for eastern areas of Drake Passage and deep regions around South Georgia (294–2,445 ind m^−3^) by Ward et al. (2007). Our data do not allow us to reveal any temporal changes in terms of integral community characteristics within the Drake Passage. In a few studies zooplankton abundances were reported to be lower, which can be explained either by sampling season (the abundance in winter months in upper water layers is lower than during spring-summer) or by different sampling methods (e.g., video recorders may underestimate the abundance) (Atkinson & Peck, 1988; Hosie et al., 2014). Stupnikova & Vereshchaka (2013) reported the biomass range from 1.2 to 65.7 mg ww m^−3^ with the highest values observed in the PF zone. In our samples biomass values were higher and varied from 13.7 to 330 mg/m^3^ (Fig. 3). However, the highest values were recorded at two stations, owing to dominance of euphausiids and fishes. After we removed these taxa from the dataset, the biomass values were reduced to 58.4 mg/m^3^, similar to the values published by Stupnikova & Vereshchaka (2013). Stupnikova et al. (2018) also reported relatively low diversity in the Drake Passage epipelagic zone compared to other areas of Southern Ocean. Shallow shelf regions around the Drake Passage, not sampled in our study, are characterized by higher abundance and biomass values comparing to deep-sea areas (Ward et al., 2007; Mackey et al., 2012).

**Table 7:** Abundance of zooplankton at different areas of Southern Ocean in relation to hydrological fronts.

**Region, Depth range (m)**	**Month**	**Position to SAF and PF**			**Reference**	**Comments**
		**SAZ**	**PFZ**	**AZ**		
Western Drake Passage, 0–250	Dec	–	752 ± 236	310 ± 295	Mackintosh (1937)	All plankton
South Georgia, 0–1,000	Jul–Aug	–	–	40	Atkinson & Peck (1988)	All plankton
Southern Drake Passage, 0–300	Dec	–	–	44 ± 36	Zmijevska (1987)	Only copepods
Near South Georgia, 0–200	Jan	–	–	1,985 ± 556	Ward et al. (1995)	All plankton
South off Africa, 1–5	Dec–Jan	538	1,738	254	Pakhomov & McQuaid (1996)	All plankton
South off New Zealand, 1–5	Jan–Feb	27	828	473	Pakhomov & McQuaid (1996)	All plankton
Near South Georgia, 0–200	Dec–Jan	–	–	294–2,445	Ward et al. (2007)	All plankton
Western Drake Passage, 10	Feb	56 ± 115	151 ± 191	80 ± 94	Takahashi et al. (2010)	Video recorder
Southern Ocean, 0–10	Nov	21	19	19	Hosie et al. (2014)	Video recorder
Drake Passage, 0–200	Oct–Nov	–	274	217	Stupnikova et al. (2018)	All plankton
Drake Passage, 0–300	Nov	845	1,126 ± 427	1,685 ± 860	This study	All plankton

**Notes.**

SAZ Subantarctic zone, north from SAF PFZ Polar Front zone, between PF and STF AZ Antarctic zone, south from PF

The values are recalculated to ind. m^−2^ and arranged by year of publication.

Biodiversity values including the Hurlbert rarefaction and Shannon–Wiener index decreased around the Polar Front in our survey, although taxonomical richness remained nearly constant (Fig. 3). Theoretically, one could expect certain increase of the biodiversity within the PF owing to polar and subpolar faunal mixing. Higher biodiversity values around the PF were previously reported from the Drake Passage (Stupnikova & Vereshchaka, 2013) and other areas of Southern Ocean (Hunt & Hosie, 2005). However, increased gradients within the PF seem to be unfavorable for many taxa and cause changes in zooplankton communities, which may be reflected in biodiversity decrease. The decrease of biodiversity in our survey could be also explained by the dominance of *Oithona* sp. copepodites contributing almost 45% to the total abundance at the PF stations (Supplemental Information).

### Taxonomical structure of zooplankton communities along the transect

A set of dominant taxa was nearly constant in our dataset with *Oithona* sp. copepodites prevailing at every station. Together with planktonic ova, these taxa contributed more than 55% of the total abundance (see Table 3). The main differences between the clusters were in proportions of *Oithona* sp. and several other taxa abundances (see Table 6). Most of the previously published investigations do not report this level of dominance, which can be explained by different sampling season and larger mesh size of the nets (Mackintosh, 1937; Atkinson & Peck, 1988; Pakhomov & McQuaid, 1996; Takahashi et al., 2010; Mackey et al., 2012). The dominance of *Oithona* sp. (identified as *Oithona similis*) was previously reported by Żmijevska (1987) and Stupnikova et al. (2018), probably due to similar sampling season. The *Oithona similis* dynamics was clearly demonstrated by Hosie et al. (2014), with a rapid increase of abundance in November and following decrease in January. Significant contribution of Ova and copepodites is a clear sign of the spring (an indication of increasing production). Apart from seasonal changes some long-term changes leading to the dominance of small copepod species (like *Oithona* spp.) were reported by Takahashi et al. (2010) in the Drake Passage. The authors compared their recent data with the results of *Discovery* Expedition described by Hardy (1936). However, the changes can be also explained by the different mesh size of the plankton nets (Takahashi et al., 2010).

Other taxa in our samples represented well known species mentioned in many investigations. No significant changes in species structure were found in this study compared to the previous investigations (Mackintosh, 1937; Voronina, 1984; Żmijevska, 1987; Pakhomov & McQuaid, 1996; Stupnikova & Vereshchaka, 2013). However, a lot of taxa were identified only to a family or class level, so we have no information on possible changes in species abundances within the major taxonomic groups (Supplemental Information).

We assumed the existence of two main boundaries along the transect represented by the SAF and the PF delimiting the zooplankton communities. Hosie et al. (2014) proposed a model predicting the spatial distribution of zooplankton communities in the Southern Ocean for each month. According to their data, the PF doesn’t act as a boundary for the surface mesoplankton communities. Instead, according to the model published, a significant boundary in the vicinity of the SAF may be expected. Our data suggest that there are two clear boundaries across the Drake Passage: first located at the SAF and the second (and more strong) at the PF. These boundaries are most conspicuous when clustering the upper 300 m layers combined (Fig. 7), but they are also visible within each of the revealed samples clusters including *Upper*, *Middle* and *Lower* clusters (Fig. 5). Species responsible for the SAF-boundary are the same in our samples and in Hosie et al. (2014), including Foraminifera and *Clausocalanus brevipes* which are more abundant south from the SAF, and *Calanoides acutus*, *Calanus propinquus*, *C. simillimus* and *Ctenocalanus* sp. which are more abundant north from the SAF (Table 6, Supplemental Information).

Dependence of plankton assemblages structure on vertical (depth) and horizontal (frontal zones) gradients along the transect is very representational (Fig. 6). Correlation of the similarity index and latitudinal distance between the stations indicates a presence of clear latitudinal gradient (Fig. 8). However, in this study the communities are more dependent on depth than on latitude or frontal zones. According to the primary data published by Żmijevska (1987) the geographical position of stations and the proximity of coast play more important role than depth within the upper 500 m. In other areas (e.g., south off Africa) the latitude gradient is far more significant than depth (Vereshchaka et al., in print). However, when only qualitative presence/absence data are considered (and all the quantitative, e.g., abundance data, excluded), epipelagic layer south of the PF formed a separate additional cluster (Fig. 6B). Therefore, the qualitative taxonomical composition is affected by both depth and frontal zones (the former is dominant), while the quantitative structure of the communities is mainly a function of depth, i.e., of trophic gradient from surface to bottom.

### The influence of temperature, salinity, depth and chlorophyll

Zooplankton in the Antarctic is known to respond to various environmental factors, including temperature, water acidification, depth, thickness of sea ice etc. (reviewed by Constable et al., 2014). The most significant factors affecting macro- and mesozooplankton distribution in the Southern Ocean are currents and fronts (Pakhomov & McQuaid, 1996). In our study this influence was less significant, partly due to peculiarity of the frontal structure in the Drake Passage (Stupnikova et al., 2018). As a result, depth was the major environmental factor influencing the spatial distribution of zooplankton, followed by salinity and temperature (see previous subsection).

In our samples notable was station 2268 creating a separate cluster (Fig. 7), probably due to the lower salinity and temperature values (Fig. 2). According to hydrological profiles this station may represent remains of a gyre or a meander. These structures frequently formed in the Drake Passage consist of waters different by salinity and/or temperature from the surrounding water masses (Olbers et al., 2004).

In contrast to previously published data (Vereshchaka et al., 2016; Vereshchaka et al., 2017; Vereshchaka, Lunina & Sutton, 2019), no significant correlations were found between total plankton biomass (in either of layers) and surface chlorophyll concentration (Tables 2 and 5). The best *R*-values were obtained for only a few taxa when analyzing the chlorophyll data for October, before the local spring bloom (see Table 2, Moore & Abbott, 2002; Demidov, Mosharov & Gagarin, 2012). The satellite chlorophyll estimation is known to be an inaccurate method comparing to direct measuring due to the ignorance of sub-surface chlorophyll and certain imperfection of algorithms. These facts may often lead to underestimations of the chlorophyll values in some areas (Garcia, Garcia & McClain, 2005; Zeng, Xu & Fischer, 2016; Brewin et al., 2017). However, despite the disadvantages of satellite chlorophyll data, our results may be a consequence of seasonal cycles of dominant zooplankton species in the Southern Ocean. Most species migrate from deep waters to the surface during different periods of biological spring and concentrate in the upper 100–200 m layer by December (Hardy & Gunther, 1935; Mackintosh, 1937; Voronina, 1984). It is likely that during the time of our survey a significant part of zooplankton remained below the sampling layers and the actual biomass was underestimated.

## Conclusions

During the early spring, the structure of zooplankton communities within the upper 1,000 m is mainly driven by depth. This is a significant factor driving quantitative composition of plankton assemblages. Another important factor is the presence of hydrological fronts. The impacts of the PF and SAF are subequal, the PF influence on the zooplankton communities is stronger, causing local increase in biomass and decrease in biodiversity. The impact of depth is greater than that of hydrological fronts if we combine all samples in a single set. The hydrological influence is mainly visible in the results of taxonomical qualitative analysis, rather than after quantitative analysis. Despite the depth impact, the effect of hydrological fronts remains conspicuous at every water layer sampled in upper 300 m. Zooplankton biomass may not be estimated by satellite chlorophyll data: robust correlations are absent, probably due to either inaccurate chlorophyll estimations or uncompleted seasonal migrations of zooplankton from deeper waters in early spring.

##  Supplemental Information

10.7717/peerj.7994/supp-1 Supplemental Information 1 Abundance and biomass of zooplankton at stations Abundances are expressed in number of individuals per cubic meter; biomass values are in gramm of wet weight per cubic meter.

## Additional Information and Declarations

### Data Availability

The following information was supplied regarding data availability: The raw data are available in File S1.
